# Supplementary material for: Silicate Inhibits the Cytosolic Influx of Chloride in Protoplasts of Wheat and Affects the Chloride Transporters, TaCLC1 and TaNPF2.4/2.5
Source: Plants (Basel). 2022 Apr 26;11(9):1162. doi: 10.3390/plants11091162 (PMC9102027; doi:10.3390/plants11091162)
Supplement: Supplementary file 1 [file plants-11-01162-s001.zip › plants-1646275-Supplementary.pdf]

(a) (b)

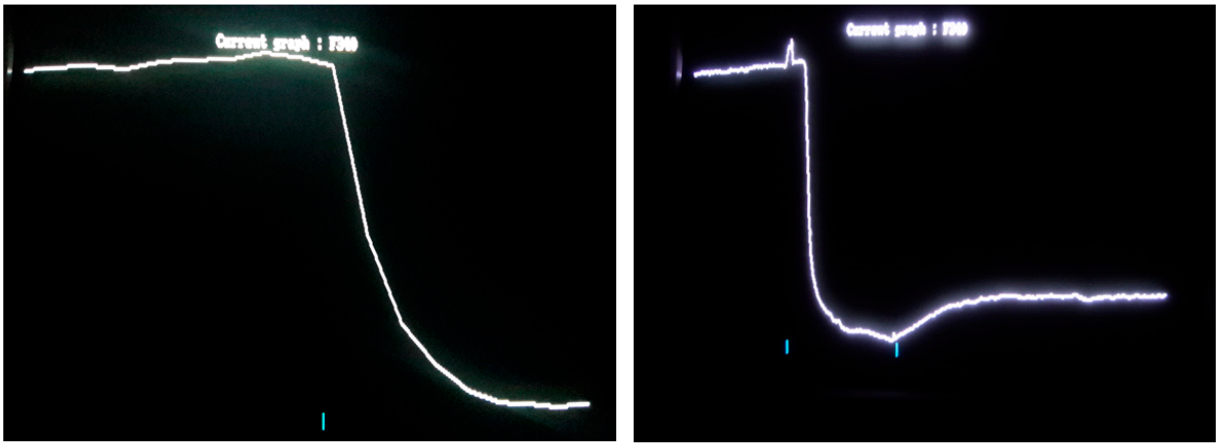

Figure S1. The original traces photographed in the computer monitor showing a decrease in fluorescence intensity after addition of 100 mM NaCl (**a**) and a gradual raise in fluorescence intensity after the addition of 1 mM K_2SiO_3 to the MQAE-labelled wheat protoplasts (**b**).

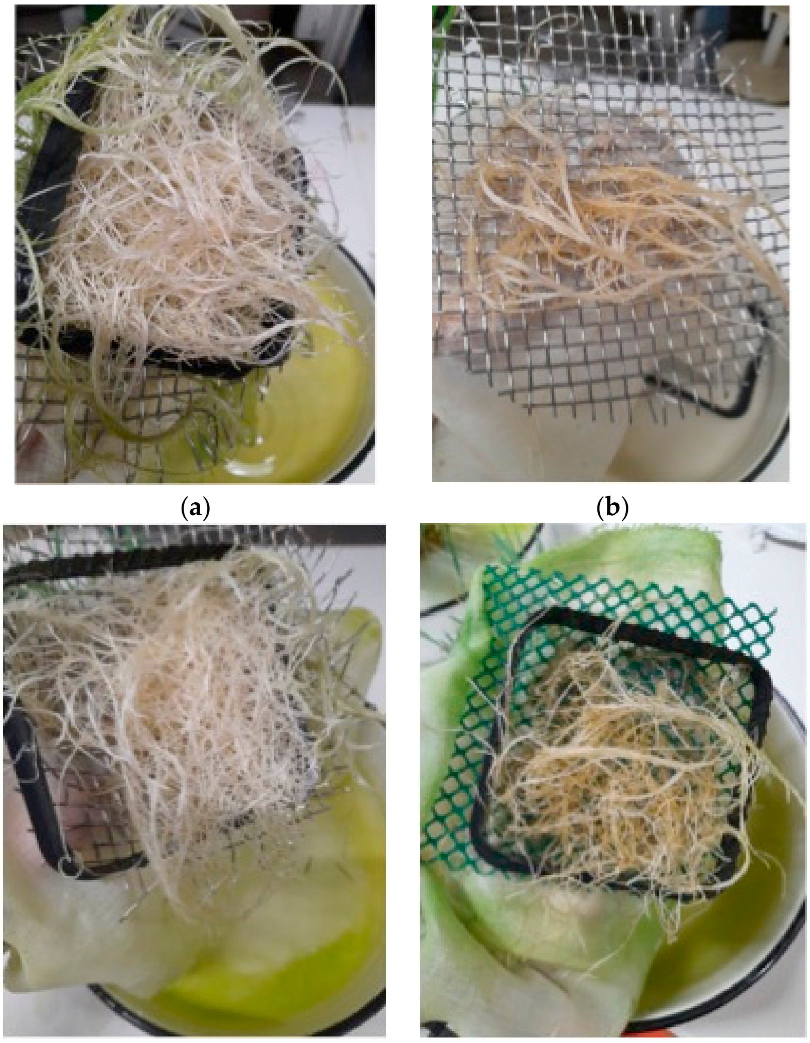

(**a**) (**b**) (**c**) (**d**)

Figure S2. Root growth of cv. Vinjett wheat seedlings grown under various treatments. (**a**) Control seedlings. (**b**) 100 mM NaCl (**c**) 1 mM K_2SiO_3 (**d**) 100 mM NaCl + 1 mM K_2SiO_3.

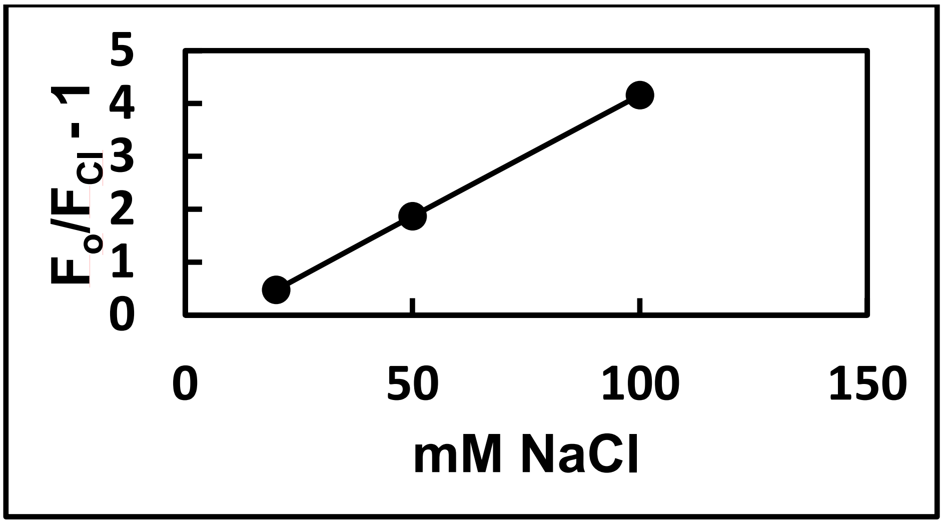

Figure S3. In situ calibration of MQAE fluorescence versus NaCl concentration shown in a Stern-Volmer plot according to Verkman et al. (1989). F_0 = fluorescence in the absence of chloride, F_{Cl} = fluorescence in the presence of Cl^-. Excitation and emission wavelengths were 350 ± 10 and 460 ± 10, respectively.
